# The independent and joint association of accelerometer-measured physical activity and sedentary time with dementia: a cohort study in the UK Biobank

**DOI:** 10.1186/s12966-023-01464-8

**Published:** 2023-05-17

**Authors:** Qi Zhong, Rui Zhou, Yi-Ning Huang, Hao-Wen Chen, Hua-Min Liu, Zhiwei Huang, Zelin Yuan, Keyi Wu, Bi-Fei Cao, Kuan Liu, Wei-Dong Fan, Yong-Qi Liang, Xian-Bo Wu

**Affiliations:** 1 Department of Epidemiology, School of Public Health, Southern Medical University (Guangdong Provincial Key Laboratory of Tropical Disease Research), No. 1063-No.1023 of Shatai South Road, Baiyun District, Guangzhou, 510515 China; 2 Department of Anaesthesiology, Nanfang Hospital, Southern Medical University, Guangzhou, China

**Keywords:** Physical activity, Sedentary time, Dementia prevention, Accelerometer

## Abstract

**Background:**

Research on the association of physical activity and sedentary time with dementia is accumulating, though elusive, and the interaction effects of the two remain unclear. We analysed the joint associations of accelerometer-measured physical activity and sedentary time with risk of incident dementia (all-cause dementia, Alzheimer’s disease and vascular dementia).

**Methods:**

A total of 90,320 individuals from the UK Biobank were included. Accelerometer-measured total volume of physical activity (TPA) and sedentary time were measured at baseline and dichotomised by median (low TPA [< 27 milli-gravity (milli-g)], high TPA [≥ 27 milli-g]; low sedentary time [< 10.7 h/day], high sedentary time [≥ 10.7 h/day]). Cox proportional hazards models were used to evaluate the joint associations with incident dementia on both additive and multiplicative scales.

**Results:**

During a median follow-up of 6.9 years, 501 cases of all-cause dementia were identified. Higher TPA was associated with a lower risk of all-cause dementia, Alzheimer’s disease and vascular dementia; the multivariate adjusted hazard ratios (HRs) (95% CI) per 10 milli-g increase were 0.63 (0.55–0.71), 0.74 (0.60–0.90) and 0.69 (0.51–0.93), respectively. Sedentary time was only found to be linked to all-cause dementia, and the HR for high sedentary time was 1.03 (1.01–1.06) compared with that for low sedentary time. No additive and multiplicative relationship of TPA and sedentary time to incident dementia was found (all *P* values > 0.05).

**Conclusion:**

Higher TPA level was related to a lower risk of incident dementia irrespective of sedentary time, which highlighted the implication of promoting physical activity participation to counteract the potential detrimental effect of sedentary time on dementia.

**Supplementary Information:**

The online version contains supplementary material available at 10.1186/s12966-023-01464-8.

## Introduction

Almost 50 million individuals worldwide are affected by dementia, one of the leading causes of death and disability adjusted life years, imposing a massive burden on patients, their caregivers, and health and social care [1, 2]. Given that the effective pharmaceutical treatments for all-cause dementia remain elusive, developing non-pharmacological strategies (i.e., lifestyle approaches) to prevent the onset of dementia is of high priority [3].

Notably, physical activity, referred to any bodily movement produced by skeletal muscles that requires energy expenditure [4], is a promising strategy for dementia prevention and disease modification [5]. A number of studies [6–11] indicated that physical activity is associated with a reduction in dementia risk. However, some studies [12, 13] have reported that physical activity might not reduce dementia risk, suggesting that physically active people have a lower risk of dementia, which can be attributed to reverse causation. Furthermore, the amount and intensity of physical activity required to prevent dementia have yet to be fully determined because a large amount of studies have relied on self-reported data [6–8, 12, 13] that are prone to recall biases and overestimation of total volume of physical activity (TPA) [14].

Sedentary behaviour, referred to any waking behaviour characterised by an energy expenditure of 1.5 METs or lower whilst sitting, reclining or lying [15], is well established on deleterious associations with health outcomes such as a higher risk of type 2 diabetes, cardiovascular disease (CVD) and all-cause mortality [16]. Accumulating evidence has indicated mixed associations between sedentary behaviours and cognitive function [17–19]. Furthermore, research into the association between sedentary behaviour and dementia risk is limited [20] and has limitations in measurement. A prospective cohort study of 431,924 UK Biobank participants [21] observed that long sedentary time is associated with higher risk of dementia. A case–control study reported that institutionalised patients with dementia spent 19.0% more time sedentary per day compared with healthy older adults [22]. The former study collected sedentary time information by using a self-reported questionnaire that only inquired about time spent watching TV and using a computer and thus cannot measure sedentary behaviour related to occupation or transportation. Furthermore, the study only targeted the specific domains of activity (watching TV and using a computer), which account for only a portion of the day. The latter study assessed sedentary behaviour by using accelerator-measured data but did not exclude the time spent on sleep, and thus its findings may be misinterpreted. Our study measured sedentary time according to the combination of accelerator-measured and self-report data, facilitated the detection and quantification of brief interruptions in sedentary behaviour and differentiation between sleep and sedation, and had a wide time scope (about a week). However, the longitudinal association of sedentary behaviour with dementia still warrants further investigation.

As regular physical activity and reduced sedentary time provide significant health benefits, current Physical Activity and Sedentary Behaviour Guidelines recommend 150–300 min of moderate intensity exercise per week, as well as limiting prolonged sedentary time [15]. However, few adults meet the physical activity recommendations, and the prevalence of sedentariness has increased over the past two decades [23, 24]. Insufficient physical activity with increased sedentary time has been shown to contribute to adverse health outcomes [25]. Nevertheless, we remain far from a complete understanding of the interrelationships between physical activity and sedentary time and their role in preserving cognitive function and reducing dementia risk. Therefore, we maximised the UK Biobank cohort and sought to investigate the independent and joint associations of accelerometer-measured TPA and sedentary time with the risk of incident dementia and its subtypes. We further investigated whether the associations of physical activity and sedentary time with dementia are affected by APOE 4-carrying status given the reported interactions between the effects of physical activity and the APOE 4, a well-known genetic risk factor for dementia and AD on dementia risk [26–29].

## Methods

### Study population

This research was conducted using the UK Biobank Resource. From 2006 to 2010, the UK Biobank recruited over 500,000 participants aged 37–73 years, with extensive phenotypic and genotypic data collected about each participant, as well as longitudinal follow-up for health outcomes [30]. All participants provided written informed consent.

**Fig. 1:**
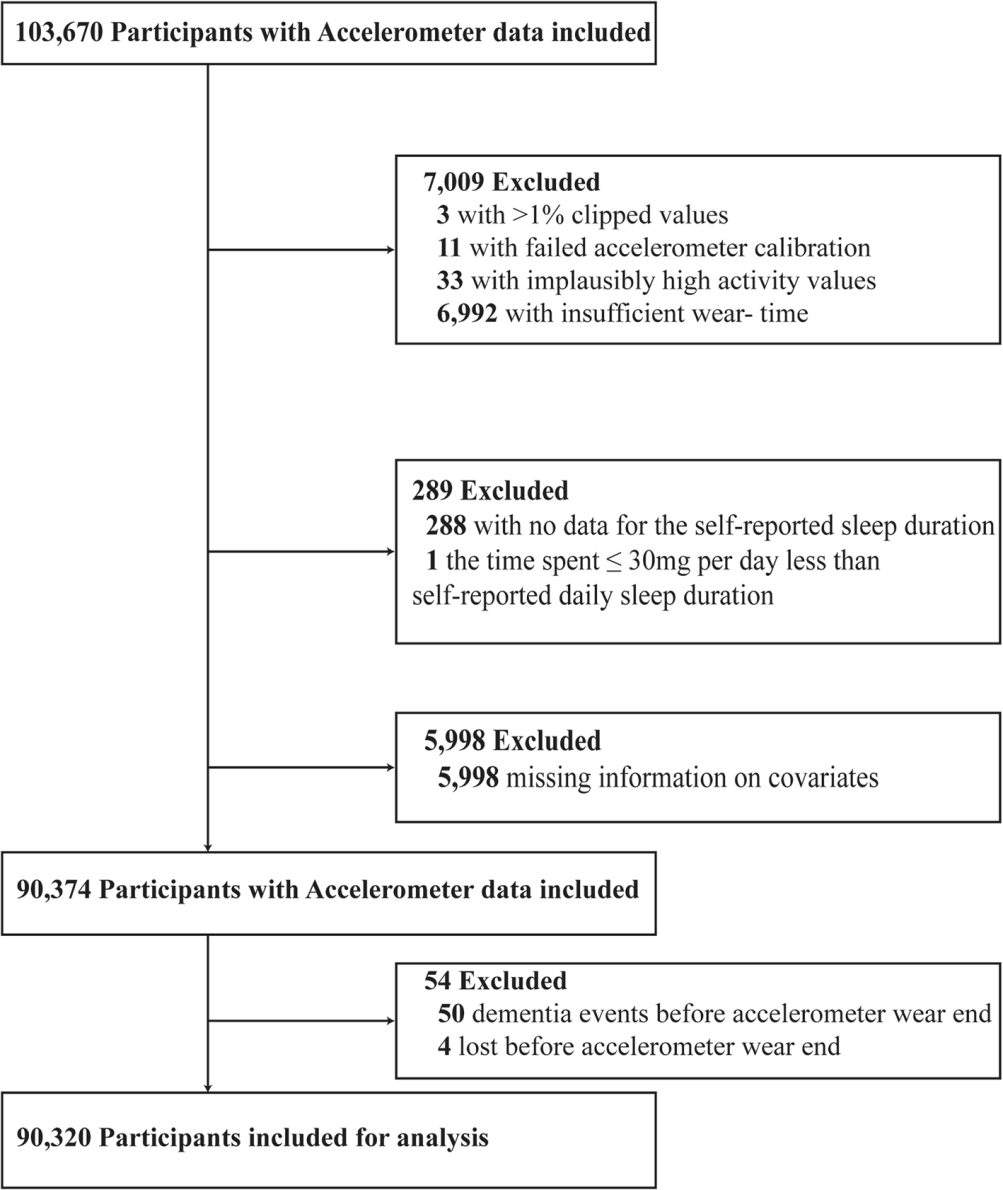
Flowchart of participant selection

Amongst 103,670 participants whose physical activity was measured with an accelerometer during May 2013 and December 2015 [31], those with failed accelerometer calibration, > 1% clipped values, implausibly high activity values or insufficient wear time were excluded (*n* = 7,009) (see Additional file 1). Furthermore, we excluded participants with missing information on self-reported sleep duration (*n* = 288), whose sleep duration exceeded the time spent on 30 milli-g per day (*n* = 1), or who had missing information concerning covariates (*n* = 5,998). Additionally, we excluded participants who had been lost to follow-up (*n* = 4) or diagnosed with dementia before the end of their accelerometer wear (*n* = 50), resulting in 90,320 participants for the final analysis (Fig. 1). Reporting of analyses and results followed the Strengthening the Reporting of Observational Studies in Epidemiology (STROBE) reporting guideline (see Additional file 2).

### Exposure assessment

Axivity AX3 wrist-worn triaxial accelerometer was used to measure TPA, with participants requested to wear the monitor continuously for 7 days on their dominant wrist [32]. TPA, a validated surrogate for global physical activity [33], was measured as the average vector magnitude in milli-gravity (milli-g) units over 7 days. Within the population distribution, we dichotomised PA by median: low TPA (< 27.0 milli-gravity [mill-g] units) and high TPA (≥ 27 mg). For ease of interpretation, we described the medians in terms of the number of minutes accumulated at an intensity equivalent to or greater than walking ‘at a brisk pace, for exercise’ (4.3 MET) [12]. This number can be predicted from the time accumulated at an acceleration above 250 milli-g [13]. The 250 mg cutoff was obtained from data from a laboratory-based calibration study [34]. Thereafter, the median (interquartile range) value of the lower median was 5.8 (4.3–10.1) minutes/day of walking at a brisk pace. Similarly, the higher median was 17.3 (13.0–25.9) minutes/day of brisk walking. Therefore, we categorised TPA in terms of low physical activity (6 min/day of brisk walking) and high physical activity (17 min/day of brisk walking) (see Additional file 3).

Self-reported sleep duration was derived from the touchscreen questionnaire at baseline. Participants were asked, ‘About how many hours of sleep do you get in every 24 h? (Please include naps)’. Participants who reported sleeping more than 12 h or less than 3 h each day were asked to confirm their answers. In addition, the answers ‘more than 23 h’ and ‘less than 1 h per day’ were rejected. To calculate the total amount of time spent in sedentary activity per day, the total amount of sleep time was subtracted from the fraction of time spent in 30 milli-g-and-under multiplied by 24. Sedentary time (h/day) were divided into binary according to median at 10.7 h/day. Thereafter, participants were categorised into four groups on the basis of combinations of binary TPA and sedentary time, with high TPA and low sedentary time as the combined reference subgroup.

### Outcome assessment

Outcomes were incident dementia and its two major component end points: Alzheimer’s disease (AD) and vascular dementia (VD), which were derived from UK Biobank’s algorithmically defined outcomes containing data obtained from baseline assessment data collection, along with linked data from hospital admissions and death registries. Detailed information about the linkage procedures can be found at elsewhere [35, 36]. The algorithms used to combine data from different sources to identify dementia have been described previously on the UK Biobank website [37]. Our outcomes were defined based on the International Classification of Diseases, 10th revision (ICD-10) and 9th revision (ICD-9) (see Additional file 4).

### Covariates

Several potential confounding factors were selected based on their known or plausible effects on physical activity and cognition [38–40]: age at baseline; sex (male and female); ethnicity (white and non-white); body mass index (BMI, underweight [< 18.5 kg/m^2^], normal weight [18.5–25 kg/m^2^], overweight [25–30 kg/m^2^] and obese [≥ 30 kg/m^2^]); smoking status (never, past or current); alcohol intake frequency (never, less than 3 times/week and $\ge$ 3 times/week); Townsend deprivation index (quintiles, with the top quintile representing most deprived); education level (high [college/university degree or above], intermediate [Advanced/Advanced Subsidiary levels, Ordinary levels, General Certificate of Secondary Education, Certificate of Secondary Education, National Vocational Qualification or Higher National Diploma, or equivalent, and other professional qualifications] and low [none of the above]). Genetic variables were calculated from apolipoprotein E (APOE) ε4 carrier status, which was determined by single nucleotide polymorphism (SNP) data for rs429358 and rs7412. Further information regarding the genotyping process can be found elsewhere (http://www.ukbiobank.ac.uk/scientists-3/genetic-data). APOE ε4 carrier status was categorised as carrying two numbers of APOE ε4 allele (genotyped ε4/ε4), 1 allele (genotyped ε2/ε4 and ε3/ε4) and none (genotyped as ε2/ε2, ε2/ε3, ε3/ε3). Health conditions included prevalence of self-reported CVD (including heart attack, angina, or stroke), hypertension, diabetes, cancer and depression assessed using the Patient Health Questionnaire (PHQ-2), with a depression score of greater than three being used to define it [41].

### Statistical analysis

Baseline characteristics by incident dementia status were compared using the $t$ test or Mann–Whitney U test for continuous variables and chi-squared tests for categorical variables.

Restricted cubic splines were used to explore the dose–effect relationship between continuous TPA and sedentary time and incident dementia risk, with five knots at its quantiles. Cox proportional hazards models were used to assess the associations between TPA and sedentary time and new onset of dementia. A priori sample size calculations for correlated data confirmed adequate power (> 80%) for the adjusted hazard ratios (aHRs) of all-cause dementia less than or equal to 0.98 (TPA) and less than or equal to 0.93 (sedentary time) [42]. The duration of follow-up was calculated as a timescale between the final date of accelerometer wear and the first event of dementia, death, loss of follow-up or censoring date (30 September 2021). On the basis of Schoenfeld residuals, the proportional hazards assumptions of the Cox model were not violated. The following models were used: model 1 was adjusted for age at baseline, sex, ethnicity, education and Townsend deprivation index; model 2 was additionally adjusted for smoking status, alcohol intake frequency, BMI, history of CVD, hypertension, diabetes, cancer and depression. The results are reported as hazard ratios (HRs) and their 95% confidence intervals (95% CIs) and incidence rate at the end of follow-up of 8 years. The adjusted Kaplan–Meier (KM) survival curve was estimated using inverse probability weighting to balance participants’ characteristics amongst the four combination groups of TPA and sedentary time [43].

The relative excess risk due to interaction (RERI) was calculated to evaluate interactions between TPA and sedentary time on an additive scale, where a RERI equals 0 implies no additive interaction and > 0 indicates a positive interaction [44]. In the present study, RERI was calculated as (${HR}_{low TPA+low SED}-{HR}_{high TPA+high SED}-{HR}_{low TPA+high SED}$)$+1$. The CI for RERI was estimated for statistical inferences by using the standard delta method [45]. Multiplicative interactions were also tested by adding a cross-product term between TPA and sedentary time in the multivariable Cox models.

Subgroup analyses were also conducted for high TPA and low sedentary time compared with low TPA and high sedentary time based on age (< 60 versus ≥ 60 years), gender (male versus female), BMI ($<$ 30 kg/m^2^ versus ≥ 30 kg/m^2^), smoking status (yes/no), baseline depression (yes/no), CVD history (yes/no) and APOE ε4 carrier status (none, one, or two). Several sensitivity analyses were also performed to assess the robustness of our study results. Firstly, to minimise reverse causality bias (i.e. undiagnosed, subclinical diseases leading to lower physical activity and dementia), we excluded participants who developed dementia within 2 years from the final date of accelerometer wear (*n* = 50). Secondly, we adjusted for APOE ε4 to exclude the confounding effect of the gene. Thirdly, the impact of missing values was assessed using multiple imputations based on 30 replications with a chained equation method in the Stata MI procedure. Additional file 5 provides detailed information on the missing variables. Fourth, we selected 9.5 h per day as the cutoff for sedentary time according to a recent meta-analysis that used device-based assessments [46]. Data were analysed using STATA 17.0 (StataCorp. 2019, College Station, TX, USA), and statistical testing was conducted at a two-tailed alpha level of 0.05.

## Results

### Subject characteristics

The baseline characteristics of the included participants are presented in Table 1. Characteristics of the excluded individuals are provided in Additional File 6.

**Table 1:** Baseline characteristics of the participants by incidence of dementia in 2006–2010

Baseline characteristic	Overall	No incidence of dementia	Incidence of Dementia	*P* value
**No. (%)**	90,320	89,819 (99.45)	501 (0.55)	
**Follow-up, years, median (IQR)**	6.9 (1.0)	6.88 (1.04)	4.93 (2.38)	< 0.001^*^
**TPA, milli-g, median (IQR)**	27.08 (10.03)	27.11 (10.03)	22.99 (8.88)	< 0.001^*^
**SED, h/day, median (IQR)**	10.71 (2.19)	10.70 (2.19)	11.06 (2.54)	< 0.001^*^
**Age at baseline, years, mean (SD)**	56.2 (7.8)	56.1 (7.8)	63.7 (5.2)	< 0.001*
**Sex, male, n (%)**	39,689 (43.94)	39,421 (43.89)	268 (53.49)	< 0.001^*^
**Ethnicity, n (%)**				0.779
White	83,586 (92.54)	83,124 (92.55)	462 (92.22)	
Non-white	6,734 (7.46)	6,695 (7.45)	39 (7.78)	
**Townsend deprivation index, n (%)**				0.251
1st quintile (least deprived)	20,828 (23.06)	20,702 (23.05)	126 (25.15)	
2nd quintile	19,699 (21.81)	19,592 (21.81)	107 (21.36)	
3rd quintile	18,635 (20.63)	18,550 (20.65)	85 (16.97)	
4th quintile	17,634 (19.52)	17,535 (19.52)	99 (19.76)	
5th quintile (most deprived)	13,524 (14.97)	13,440 (14.96)	84 (16.77)	
**Education, n (%)**				< 0.001^*^
Low	7,205 (7.98)	7,112 (7.92)	93 (18.56)	
Intermediate	43,333 (47.98)	43,119 (48.01)	214 (42.71)	
High	39,782 (44.05)	39,588 (44.08)	194 (38.72)	
**Smoking status, n (%)**				< 0.001^*^
Never	51,639 (57.17)	51,402 (57.23)	237 (47.31)	
Previous	32,383 (35.96)	32,251 (35.91)	232 (46.31)	
Current	6,198 (6.86)	6,166 (6.86)	32 (6.39)	
**Alcohol intake frequency, n (%)**				< 0.001^*^
Never	5,011 (5.55)	4,964 (5.53)	47 (9.38)	
Less than 3 times/week	40,677 (45.04)	40,476 (45.06)	201 (40.12)	
≥ 3 times/week	44,632 (49.42)	44,379 (49.41)	253 (50.50)	
**BMI category (kg/m**^**2**^**), n (%)**				0.155
Underweight (< 18.5)	503 (0.56)	502 (0.56)	1 (0.20)	
Normal weight (18.5–25)	35,187 (38.96)	34,991 (38.96)	196 (39.12)	
Overweight (25–30)	37,256 (41.25)	37,065 (41.27)	191 (38.12)	
Obese (≥ 30)	17,374 (19.24)	17,261 (19.22)	113 (22.55)	
**CVD event, n (%)**	3,488 (3.86)	3,426 (3.81)	62 (12.38)	< 0.001^*^
**Hypertension, n (%)**	20,612 (22.82)	20,439 (22.76)	173 (34.53)	< 0.001^*^
**Diabetes, n (%)**	3,066 (3.39)	3,017 (3.36)	49 (9.78)	< 0.001^*^
**Cancer, n (%)**	6,620 (7.33)	6,579 (7.32)	41 (8.18)	0.462
**Depression, n (%)**	3,476 (3.85)	3,454 (3.85)	22 (4.39)	0.527
**APOE ε4 carrier status, n (%)**				< 0.001^*^
ε4 non-carrier	54,196 (60.00)	53,995 (60.12)	201 (40.12)	
One ε4 allele	19,582 (21.68)	19,406 (21.61)	176 (35.13)	
Two ε4 alleles	1,664 (1.84)	1,627 (1.81)	37 (7.39)	
Missing data, No. (%)	14,878 (16.47)	14,791 (16.47)	87 (17.37)	

*Abbreviations*: *TPA* total volume of physical activity, milli-g, *BMI* body mass index, *CVD* cardiovascular disease, *IQR* interquartile range, *SD* standard deviation

^*^P<0.05

Of the 90,320 participants, the mean (SD) age was 56.2 (7.8) years, and 39,689 (44%) were males. During a median follow-up of 6.9 person years, 501 participants (0.55%) developed dementia (199 cases of AD and 94 cases of VD). Participants with dementia were more likely to be older; male; previous or current smokers; APOE ε4 carriers; with a lower education level and alcohol intake frequency; and had a higher prevalence of CVD, hypertension and diabetes.

### Nonlinear association

**Fig. 2:**
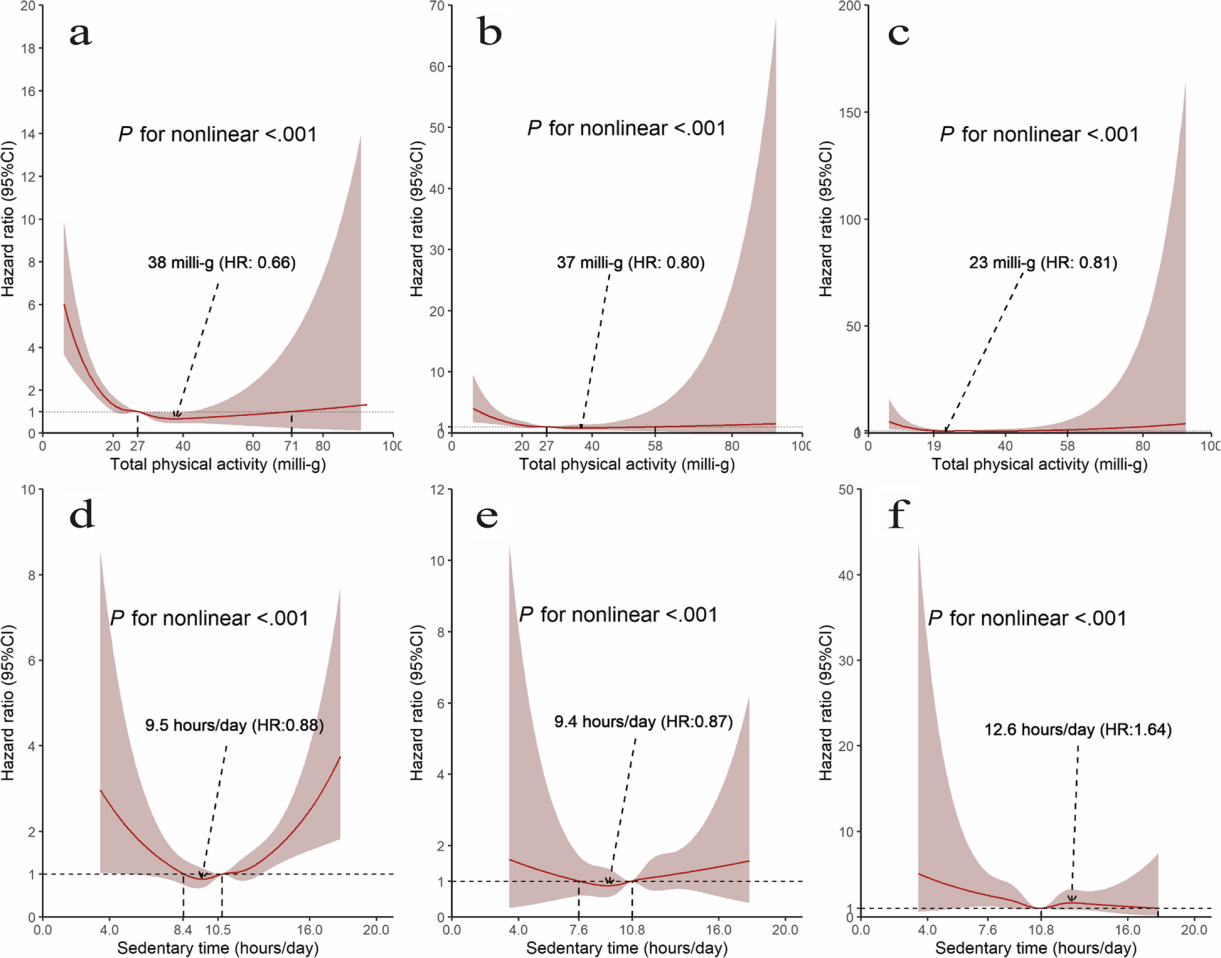
Restricted cubic splines for dose–response associations between TPA and sedentary time with incident dementia. **a** TPA and all-cause dementia; **b** TPA and Alzheimer’s disease; **c** TPA and vascular dementia; **d **Sedentary time and all-cause dementia; **e** Sedentary time and Alzheimer’s disease; **f** Sedentary time and vascular dementia. The 95% CIs of the adjusted hazard ratios are represented by the shaded area. Multivariable models were adjusted for age at baseline, sex, ethnicity, education and Townsend deprivation index, smoking status, alcohol intake frequency, body mass index, baseline cardiovascular disease, hypertension, diabetes, cancer and depression

In multivariate-adjusted models (Fig. 2), the restricted cubic splines showed that the associations of TPA and sedentary time with dementia and its subtypes were nonlinear (*P*s for nonlinear < 0.001). Furthermore, a reverse relationship between TPA and risk of all-cause dementia (*P* < 0.001) was observed up to doses of around 38 milli-g and then a flat to increasing trend as the volume rose to 100 milli-g. Similar patterns to those reported for all-cause dementia were observed for AD and VD. As for sedentary time, the U‑shaped associations for all-cause dementia and AD were observed with a nadir at approximately 9.5 and 9.4 h/day, respectively.

### Effect of TPA and sedentary time on dementia

In the unadjusted Cox model Table 2, higher TPA was significantly associated with lower risk of incident all-cause dementia, AD and VD. These associations were slightly attenuated in magnitude but remained significant after multivariable adjustment. For each 10 milli-g increase in TPA, the HRs of all-cause dementia, AD and VD were 0.63 (95% CI: 0.55–0.71), 0.74 (0.60–0.90) and 0.69 (0.51–0.93), respectively. For the sedentary time analyses, there was only a positive association with all-cause dementia (HR: 1.22; 95% CI: 1.02–1.47). The HR per 0.5 h increase in sedentary time was 1.03 (95% CI: 1.01–1.06) for the incidence of all-cause dementia.

**Table 2:** Association of TPA and sedentary time with incident dementia and its subtypes

	**Events/N**	**Incidence rate (per 1000 person-years)**	**Crude model**		**Model 1**		**Model 2**	
			**HR (95% CI)**	***P* value**	**HR (95% CI)**	***P* value**	**HR (95% CI)**	***P* value**
**All-cause dementia**								
**TPA, milli-g**								
Low	369/45180	1.20	Ref		Ref		Ref	
High	132/45140	0.43	**0.35 (0.29, 0.43)**	** < 0.001**	**0.57 (0.47, 0.70)**	** < 0.001**	**0.58 (0.47, 0.71)**	** < 0.001**
Continuous (per 10 mg)	501/90,320	0.81	**0.44 (0.39, 0.50)**	** < 0.001**	**0.62 (0.55, 0.70)**	** < 0.001**	**0.63 (0.55, 0.71)**	** < 0.001**
**Sedentary time, h/ day**								
Low	205/45,439	0.66	Ref		Ref		Ref	
High	296/44,881	0.97	**1.48 (1.24, 1.77)**	** < 0.001**	**1.24 (1.03, 1.48)**	**0.020**	**1.22 (1.02, 1.47)**	**0.032**
Continuous (per 0.5 h)	501/90,320	0.81	**1.08 (1.05, 1.10)**	** < 0.001**	**1.04 (1.01, 1.07)**	**0.004**	**1.03 (1.01, 1.06)**	**0.014**
**Alzheimer’s disease**								
**TPA, milli-g**								
Low	143/45180	0.47	Ref		Ref		Ref	
High	56/45,180	0.18	**0.39 (0.28, 0.53)**	** < 0.001**	**0.65 (0.47, 0.89)**	** < 0.007**	**0.63 (0.45, 0.87)**	**0.004**
Continuous (per 10 mg)	199/90,320	0.32	**0.53 (0.44, 0.64)**	** < 0.001**	**0.76 (0.62, 0.92)**	**0.004**	**0.74 (0.60, 0.90)**	**0.003**
**Sedentary time, h/ day**								
Low	86/45439	0.28	Ref		Ref		Ref	
High	113/44881	0.37	**1.34 (1.01, 1.78)**	**0.039**	1.14 (0.86, 1.51)	0.366	1.16 (0.87, 1.55)	0.305
Continuous (per 0.5 h)	199/90,320	0.32	**1.05 (1.01, 1.10)**	**0.014**	1.02 (0.98, 1.06)	0.342	1.02 (0.98, 1.07)	0.319
**Vascular dementia**								
**TPA, milli-g**								
Low	63/45180	0.21	Ref		Ref		Ref	
High	31/45140	0.09	**0.48 (0.31, 0.74)**	**0.001**	0.84 (0.54, 1.29)	0.421	0.93 (0.59, 1.47)	0.759
Continuous (per 10 mg)	94/90,320	0.15	**0.43 (0.33, 0.57)**	** < 0.001**	**0.63 (0.48, 0.85)**	**0.002**	**0.69 (0.51, 0.93)**	**0.013**
**Sedentary time, h/ day**								
Low	45/45439	0.14	Ref		Ref		Ref	
High	49/44881	0.16	1.12 (0.74, 1.67)	0.597	0.89 (0.59, 1.34)	0.565	0.83 (0.55, 1.27)	0.392
Continuous (per 0.5 h)	94/90,320	0.15	1.03 (0.97, 1.09)	0.403	0.98 (0.93, 1.05)	0.606	0.97 (0.91, 1.03)	0.288

Model 1 adjusted for age at baseline, sex, ethnicity, education and Townsend deprivation index

Model 2 additionally adjusted for smoking status, alcohol intake frequency, body mass index, baseline cardiovascular disease, hypertension, diabetes, cancer and depression

*Abbreviations*: *TPA* total volume of physical activity, milli-g

### Joint effect of TPA and sedentary time on dementia

**Fig. 3:**
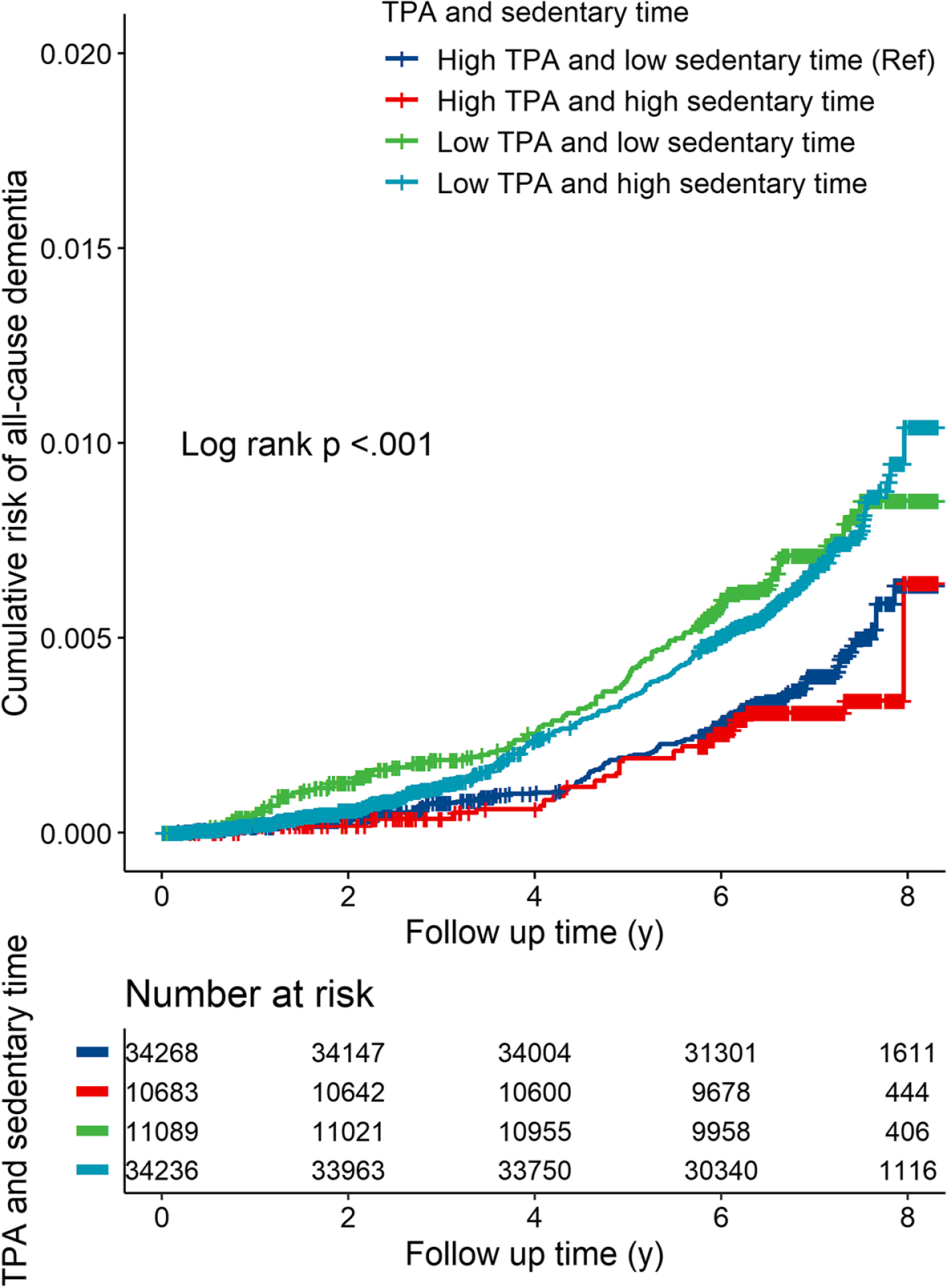
Adjusted risk of incident dementia according to TPA (milli-g) and sedentary time (h/day) profile. Cox proportional hazards models were adjusted for age at baseline, sex, ethnicity, education and Townsend deprivation index, smoking status, alcohol intake frequency, body mass index (BMI), history of cardiovascular disease (CVD), hypertension, diabetes, cancer and depression. Abbreviations: TPA, total volume of physical activity; milli-g

The adjusted cumulative risks of all-cause dementia stratified by TPA and sedentary time categories are shown in Fig. 3, which revealed a log-rank *P* < 0.001. Table 2 shows the joint associations of TPA and sedentary time with incident dementia risk. Compared with those with high TPA and low sedentary time, participants with low TPA and high sedentary time had significantly higher risk of all-cause dementia and AD, with multivariate-adjusted HRs of 1.65 (95% CI: 1.31–2.09, *P* < 0.05) and 1.51 (95% CI: 1.06–2.17, *P* < 0.05), respectively. The detrimental HR of high sedentary time was more evident amongst participants with low TPA, though no clear interactions were observed between TPA and sedentary time on either additive (all *P* values for RERI were > 0.05) or multiplicative scales (*P* for interaction > 0.05; Table 3).

**Table 3:** Additive and multiplicative interaction effects between TPA and sedentary time on the dementia incidence

TPA, milli-g	Sedentary time, hours/ day	Events/N	Incidence rate (per 1000 person-years)	HR (95% CI)	*P* value	Additive interaction (RERI)		Multiplicative interaction	
						**Estimates (95% CI)**	***P* value**	**Estimates (95% CI)**	***P* value**
**All-cause dementia**						0.25 (− 0.26, 0.77)	0.335	1.31 (0.79, 2.18)	0.295
High	Low	109/34371	0.46	Ref					
High	High	23/10769	0.31	0.78 (0.50, 1.22)	0.274				
Low	Low	96/11068	1.28	**1.62 (1.23, 2.15)**	**0.001**				
Low	High	273/34112	1.18	**1.65 (1.31, 2.09)**	** < 0.001**				
**Alzheimer’s disease**						0.26 (− 0.51, 1.03)	0.502	1.42 (0.64, 3.17)	0.391
High	Low	47/34371	0.20	Ref					
High	High	9/10769	0.12	0.74 (0.36, 1.52)	0.413				
Low	Low	39/11068	0.52	1.51 (0.98, 2.32)	0.062				
Low	High	104/34112	0.45	**1.51 (1.06, 2.17)**	**0.024**				
**Vascular dementia**						0.33 (− 0.48, 1.13)	0.427	1.74 (0.53, 5.69)	0.359
High	Low	27/34371	0.11	Ref					
High	High	4/10,769	0.05	0.52 (0.18, 1.50)	0.226				
Low	Low	18/11068	0.24	1.07 (0.58, 1.97)	0.828				
Low	High	45/34112	0.19	0.92 (0.55, 1.52)	0.738				

Models were adjusted for age at baseline, sex, ethnicity, education and Townsend deprivation index, smoking status, alcohol intake frequency, body mass index (BMI), history of cardiovascular disease (CVD), hypertension, diabetes, cancer and depression

*Abbreviations*: *TPA*, total volume of physical activity; milli-g, *RERI* relative excess risk due to interaction

### Additional analyses

**Fig. 4:**
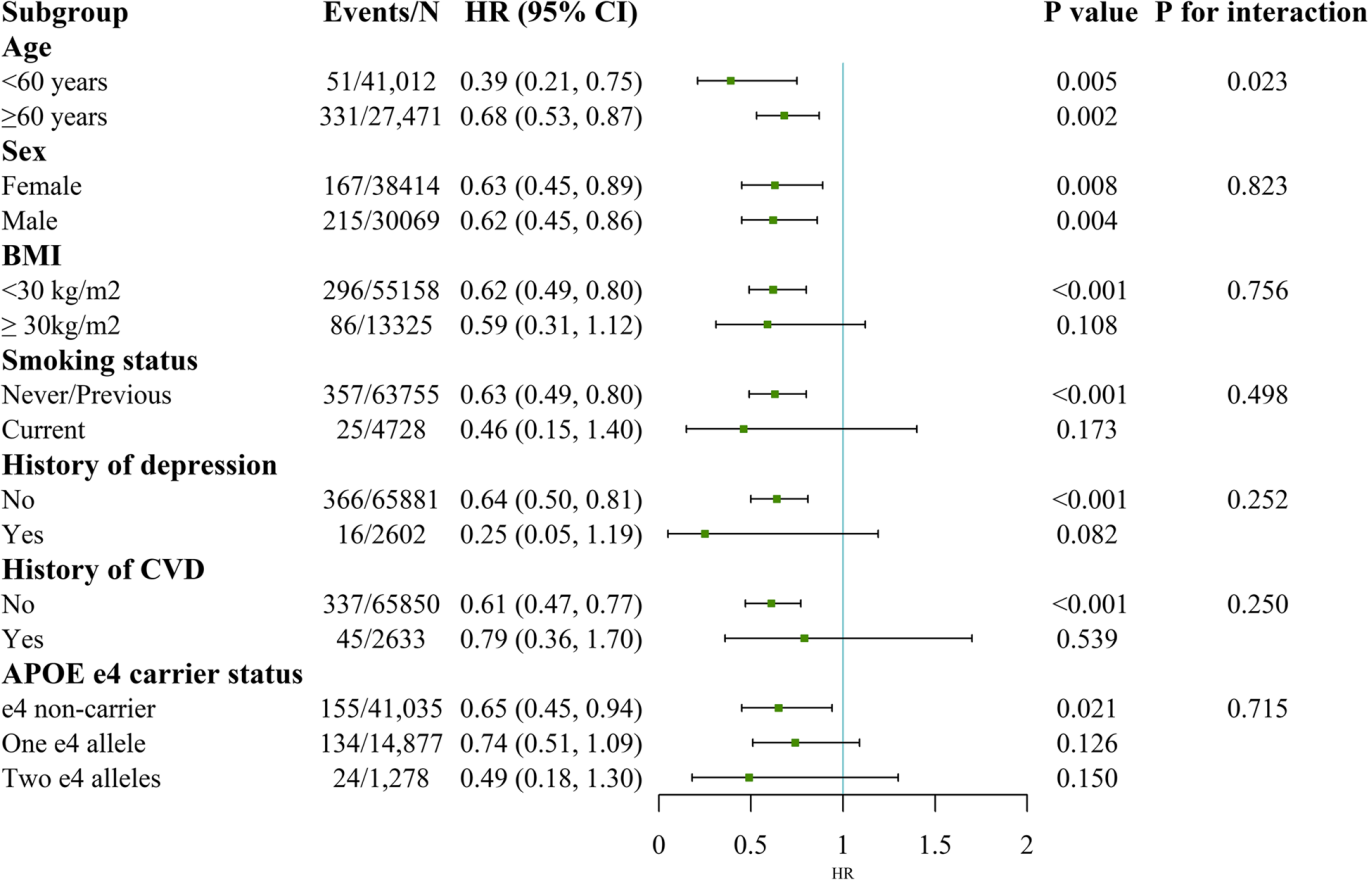
Stratified analysis for the association between TPA and sedentary time with all-cause dementia risk. Subgroup analyses were conducted for high TPA and low sedentary time compared with low TPA and high sedentary time. Models were adjusted for age at baseline, sex, ethnicity, education, Townsend deprivation index, smoking status, alcohol intake frequency, body mass index, history of cardiovascular disease, hypertension, diabetes, cancer and depression. Abbreviations: TPA, total volume of physical activity, milli-g; BMI, body mass index; CVD, cardiovascular disease

**Fig. 5:**
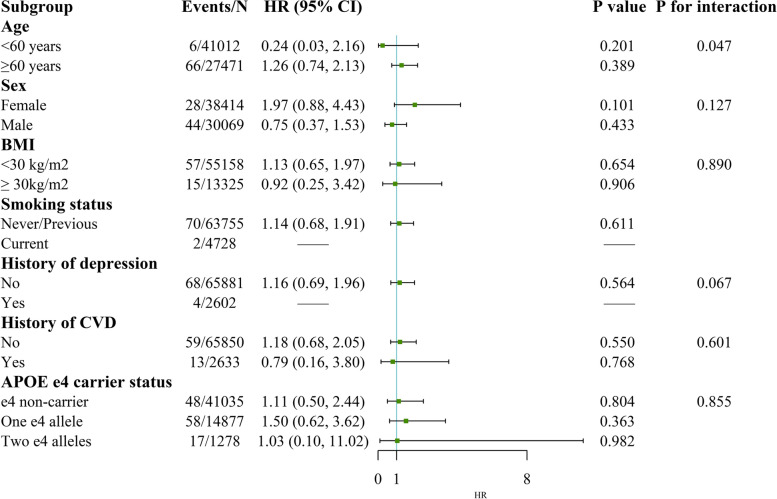
Stratified analysis for the association between TPA and sedentary time with vascular dementia risk. Subgroup analyses were conducted for high TPA and low sedentary time compared with low TPA and high sedentary time. Models were adjusted for age at baseline, sex, ethnicity, education, Townsend deprivation index, smoking status, alcohol intake frequency, body mass index, history of cardiovascular disease, hypertension, diabetes, cancer and depression. Abbreviations: TPA, total volume of physical activity, milli-g; BMI, body mass index; CVD, cardiovascular disease

**Fig. 6:**
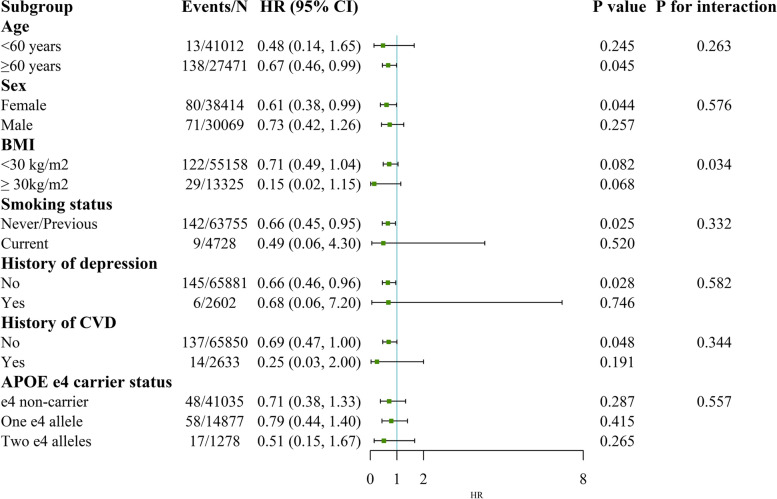
Stratified analysis for the association between TPA and sedentary time with Alzheimer’s disease risk. Subgroup analyses were conducted for high TPA and low sedentary time compared with low TPA and high sedentary time. Models were adjusted for age at baseline, sex, ethnicity, education, Townsend deprivation index, smoking status, alcohol intake frequency, body mass index, history of cardiovascular disease, hypertension, diabetes, cancer and depression. Abbreviations: TPA, total volume of physical activity, milli-g; BMI, body mass index; CVD, cardiovascular disease

The association of high-TPA and low sedentary time with the risk of all-cause dementia and VD was pronounced in individuals aged below 60 years (*P* values for interaction were 0.023 and 0.047, respectively, see Figs. 4 and 5). Regarding the subgroup analyses of the HRs of AD, we found significantly modified effects for BMI (*P* for interaction = 0.034, see Fig. 6). The results were unchanged after performing several sensitivity analyses (Additional files 7, 8, 9 and 10).

## Discussion

Using a large prospective cohort study, we found that TPA was associated with lower risk of all-cause dementia and AD but not with VD; sedentary time was associated with higher risk of all-cause dementia but not its subtypes. More importantly, the risks of all-cause dementia further increased amongst participants with both low TPA and high sedentary time, although no additive or multiplicative interactions were observed. Our findings remained robust after performing several sensitivity and stratified analyses.

### Comparison with other studies

Physical activity and dementia risk have been investigated in several epidemiological studies. According to a previous longitudinal study in middle-aged women, physical activity was found to be associated with a low risk of all-cause dementia over 44 years but not for AD or VD [7]. However, over a mean follow-up of 26.6 years, the Whitehall II cohort study found no association between TPA and dementia [13]. Reasons for such discrepancies could be a variety of factors, such as follow-up time, demographic background, adjustment for confounders and assessment of physical activity. In these prospective studies, TPA was measured by a self-reported questionnaire, which may prevent capturing leisure or non-leisure physical activity across multiple domains, leading to recall bias [47]. The findings in our study contribute to evidence linking accelerometer-measured TPA with decreased risk of incident all-cause dementia, Alzheimer’s disease and vascular dementia. However, there remained issues with their understanding of the domain, context and purposes of physical activity. Therefore, self-reported and device-based methods should be combined to provide a complete picture. The findings for detrimental effects of sedentary time on dementia and cognition have been inconsistent. Our findings supported that sedentary time was related to high risk of all-cause dementia, in accordance with a previous meta-analysis [48] that included 18 cohort studies involving 250,063 participants. Nevertheless, a coordinated analysis across five cohort studies [17] suggested that sedentary time was not related to global cognition in elder adults. Its inconsistency could be partly explained by its insufficient sample size (*n* = 10,450), different follow-up times, different demographics and the way dementia and sedentary time are measured and defined. In the current study, the large sample size (*n* = 90,320) afforded considerable statistical power. In addition, the likelihood of reverse causality was minimised by adjusting for a wide range of covariates.

Our study found that the combination of lower TPA and higher sedentary time indicated an increased risk of incident all-cause dementia and AD. However, our study found no interaction between TPA and sedentary time that was associated with all-cause dementia and its subtypes. As confirmed by Raichlen and colleagues’ previous study [49], leisure sedentary time is associated with all-cause dementia risk regardless of physical activity level, with some attenuation at high levels of physical activity. This suggested two distinct behavioural pathways that can alter dementia risk. Huang and colleagues [21] also reported that high leisure-time physical activity is associated with low dementia risk even amongst high sedentary time groups. Nevertheless, the potential interaction between TPA and sedentary time on the subsequent risk of dementia was not considered in both of them. Given the importance of interaction assessment for identifying biological mechanisms (e.g. synergism or antagonism between two exposures) and improving preventive interventions [44], our study added new evidence about joint associations by exploring the interaction on both additive and multiplicative scales.

Furthermore, our study observed that the association between the combination of high TPA and low sedentary time and the risk of all-cause dementia and VD was more pronounced in people < 60 years, whereas the association with AD was stronger in obese participants (BMI > 30 kg/m^2^). This finding can be partly explained by the global surveillance of physical activity [50] that younger adults (18–24 years) are more physically active than older adults (≥ 75 years), which may contribute to more beneficial gains. In addition, the ageing brain is vulnerable to a variety of cognition-impairing neuropathologies (e.g. cerebrovascular conditions) [51], which may contribute to a high risk of dementia in the elderly. Implementing early interventions focused on improving physical activity and reducing sedentary time for cognitive decline at midlife is a strategy with a high chance of success. Additionally, our findings regarding AD and physical activity in midlife were consistent with those of two previous studies [7, 52]. These studies found no significant association between physical activity in midlife and subsequent development of AD. Regarding the BMI-specific difference observed in AD, we speculated that physical activity may mitigate the negative impact of obesity on AD by modifying the association between obesity and reduced cerebral blood flow [53]. Physical activity may contribute to weight loss, which is associated with a high risk of later-life dementia amongst middle-aged and older adults [54].

### Potential mechanism

Several mechanisms whereby physical activity may be related to dementia have been proposed [55, 56]. Firstly, physical activity has proven to be beneficial for traditional cardiovascular risk factors (e.g. reduced vascular flow and diabetes) involved in dementia pathogenesis. Secondly, physical activity also promotes neurogenesis via increases in exercise-induced metabolic factors (e.g. ketone bodies and lactate) and muscle-derived myokines (cathepsin-B and irisin), which in turn stimulate the production of neurotrophins such as brain-derived neurotrophic factor. Finally, physical activity exerts anti-inflammatory effects and improves the brain redox status, thereby ameliorating amyloid-β deposition. However, sedentary time and cognitive health are less well understood biologically. Sedentary time contributes to CVD by increasing systemic inflammation; reducing blood flow and shear stress; and increasing blood pressure, postprandial glucose, insulin and triacylglycerol [57]. Thus, the increasing evidence for the association between cardiovascular health and dementia [58] may be a pathway between sedentary time and incident dementia. Further studies are warranted to elucidate the biological mechanisms of sedentary time and dementia.

In our study, sedentary time level appeared to modify the magnitude of the associations between TPA and incident dementia. This finding could be interpreted as a reinforcement that the importance of physical activity increases as people become more sedentary. Given the role of brain insulin resistance in the development and progress of AD, cognitive function and memory [59], we speculated that the deleterious consequences of prolonged sedentary time can be mitigated with short bouts of physical activity [60]. Considering the absence of interaction effects between TPA and sedentary time, sedentary time likely influences the risk of dementia incidence in part through distinct mechanisms that act independent of physical activity. The beneficial effects from physical activity might overweigh the limited risks of sedentary time. In support of our findings, studies reported that the effects of increasing physical activity on CVD and diabetes mellitus can be mediated centrally through the brain, and the metabolic and vascular consequences of inadequate physical activity appear to be mediated primarily through peripheral tissues and cells, including muscle, adipose tissue, and endothelial and inflammatory cells [60]. However, whether these biological mechanisms are related to dementia has not yet been demonstrated. Regarding the little evidence for the interaction effect on neurodegeneration diseases, further studies on the pathophysiological changes are highly warranted.

### Strengths and limitations of this study

Amongst the strengths of this study were its large sample size of UK Biobank participants, device-based physical activity, prospective design, comprehensive outcome (including dementia and its subtypes) and collection of various potential confounders (such as APOE ε4 carrier status). Nevertheless, our study also had some limitations. First, 13,350 participants were excluded from the present study, who were more likely to be current smokers, obese and had lower education level than those enrolled in the study. Thus, the incidence of dementia in our current study may be underestimated. Second, device-based methods are limited in the horizontal locomotion and unable to distinguish between types of physical activity and sedentary behaviour. Future research combining accelerometer-measured and self-reported methods is needed to verify our findings. Future research combining accelemeter-measured and self-reported methods is needed to verify our findings. Third, this study could not establish a causal relationship given the observational study design. However, our findings remained largely unchanged after excluding participants who developed dementia within 2 years, which made our results more convincing. Fourth, using TPA as a proxy for global physical activity can be a limitation that incapable explain the benefit of physical activity intensity to cognitive function, which was acknowledged. Fifth, a median value of 10.7 h per day for sedentary time in the present study was greater than the cutoff value employed in other studies to differentiate between high and low sedentary time. Thus, the generalizability of our findings is limited. Further studies are warranted using diverse populations with sedentary time and distinct sedentary behaviors measured. Finally, the UK Biobank participants is ethnically and racially homogeneous, leading to limitations for generalizability, though researchers have argued that results from this cohort could be externally valid for linking exposures with health outcomes [61].

## Conclusion

In conclusion, we found that higher physical activity and lower sedentary time were independently associated with lower risk of all-cause dementia, and the risks were augmented when they were present together. Moreover, no interaction effects between TPA and sedentary time on dementia were observed, indicating that physical activity may be considered a potentially protective factor irrespective of sedentary time against dementia. The findings also illustrated the significance of increasing participation in physical activity, and controlling sedentary time should be prioritised throughout one’s lifetime to prevent dementia incidence.

## Supplementary Information

**Additional file 1.** Definition of the accelerometer data quality.

**Additional file 2.** STROBE Statement—Checklist of items that should be included in reports of cohort studies.

**Additional file 3.** Methods supplement for the measurement of total volume of physical activity.

**Additional file 4.** Codes used in the UK Biobank study to identify dementia cases.

**Additional file 5.** Detailed information on missing covariates.

**Additional file 6.** Baseline characteristics of excluded individuals.

**Additional file 7.** Association of TPA and sedentary time with dementia excluding dementia onset within 2 years (*n* = 47).

**Additional file 8.** Association of TPA and sedentary time with dementia after additional adjustment for APOE ε4.

**Additional file 9.** Association of TPA and sedentary time with the incident dementia risk (multiple imputation).

**Additional file 10. **Association of TPA and sedentary time with dementia using a 9.5-h cutoff for sedentary time.

## Data Availability

The data of this study can be requested from the UK Biobank (https://www.ukbiobank.ac.uk/). This work was conducted under UK Biobank application number 55794.

## Abbreviations

TPA: Total physical activity
CVD: Cardiovascular disease
AD: Alzheimer’s disease
VD: Vascular dementia
ICD-9: The International Classification of Diseases 9th revision
ICD-10: The International Classification of Diseases 10th revision
BMI: Body mass index
APOE: Apolipoprotein E
RERI: The relative excess risk due to interaction
HR: Hazard ratio
CI: Confidence interval
UK Biobank: United Kingdom Biobank
