# Supplementary material for: The independent and joint association of accelerometer-measured physical activity and sedentary time with dementia: a cohort study in the UK Biobank
Source: Int J Behav Nutr Phys Act. 2023 May 17;20:59. doi: 10.1186/s12966-023-01464-8 (PMC10190060; doi:10.1186/s12966-023-01464-8)
Supplement: Supplementary file 1 — Additional file 1. Definition of the accelerometer data quality. [file 12966_2023_1464_MOESM1_ESM.docx]

**Additional File 1.** Definition of the accelerometer data quality.

| **Data quality** | **Definition** | **UK Biobank Field ID** |
| --- | --- | --- |
| Insufficient wear-time | Insufficient wear time indicated the unavailability of at least 72 hours of data or lacked data for every 1-hour period of the 24-hour cycle (scattered over multiple days). | 90015 |
| Implausibly high activity values | Implausibly high activity values defined as average vector magnitude scores of >100 milli-gravity. | 90012 |
| Clipped values | The Axivity device was set up to capture tri-axial acceleration data over a seven day period at 100Hz with a dynamic range of ±8 g.  Clipped values, which occur when the sensor's dynamic range of ±8 g is exceeded, were flagged before and after calibration.  We calculated the percentage of clipped values as readings exceeding ±8 g before (or after) calibration divided by total data readings and multiplyed 100%, and excluded those with clipped values >1%. | 90183, 90185, 90187 |
| Calibration | The acceleration signals were failed to be calibrated to local gravity due to insufficient data were available for a given participant (or if unavailable, the previous participant) where any of the three sensor axes did not have values outside a +- 300 mg range. We used calibrated acceleration data to ensure different devices provided comparable data outputs. | 90016 |
