# Supplementary material for: The independent and joint association of accelerometer-measured physical activity and sedentary time with dementia: a cohort study in the UK Biobank
Source: Int J Behav Nutr Phys Act. 2023 May 17;20:59. doi: 10.1186/s12966-023-01464-8 (PMC10190060; doi:10.1186/s12966-023-01464-8)
Supplement: Supplementary file 3 — Additional file 3. Methods supplement for the measurement of total volume of physical activity. [file 12966_2023_1464_MOESM3_ESM.docx]

**Additional File 3.** Methods supplement for the measurement of the total volume of physical activity

We have used the overall acceleration average given that this measures the total time spent in different levels of PA intensity and has previously demonstrated a high correlation with total energy expenditure ([1](#_ENREF_1), [2](#_ENREF_2)). Within the population distribution, we dichotomised PA by median: low TPA (<27.0 milli-gravity [mill-g] units) and high TPA (≥27 mg). For ease of interpretation, we described the medians in terms of the number of minutes accumulated at an intensity equivalent to or greater than walking ‘at a brisk pace, for exercise’ (4.3 MET) ([3](#_ENREF_3)). This number can be predicted from the time accumulated at an acceleration above 250 milli-g ([4](#_ENREF_4)). The 250 mg cutoff was obtained from data from a laboratory-based calibration study ([4](#_ENREF_4)). This study included 30 adults aged 18–65 years and the following activities: lying, sitting, standing, lifestyle activities, slow walking, fast walking, running and walking up and down a step. We translated activity levels in terms of brisk walking as it is a moderate-intensity activity frequently cited as an example in physical activity guidelines (e.g. https://www.nhs.uk/live-well/exercise/). The energy expenditure compendium reports 4.3 MET is indicative of walking at a ‘brisk pace for exercise’ ([3](#_ENREF_3)). The regression equation presented by the authors of the calibration study for wrist acceleration and energy expenditure in adults showed that an acceleration of 250 mg predicted 4.3 MET. We used the fraction time ≤250 mg acceleration to carry out the calculation. The median (interquartile range) value of the lower median was 5.8 (4.3–10.1) minutes/day of walking at a brisk pace. Similarly, the higher median was 17.3 (13.0–25.9) minutes/day of brisk walking. Therefore, for ease of interpretation, we categorised TPA in terms of low physical activity (6 minutes/day of brisk walking) and high physical activity (17 minutes/day of brisk walking). To ensure our results were valid, we carried out sensitivity analyses and based the medians only on the population who spent time above 250 mg. The median (interquartile range) value of the lower median was 7.2 (4.3–8.6) minutes/day, and the high median was 20.2 (15.8–27.4) minutes/day of brisk walking.

**Reference:**

1. van Hees VT, Renström F, Wright A, Gradmark A, Catt M, Chen KY, et al. Estimation of daily energy expenditure in pregnant and non-pregnant women using a wrist-worn tri-axial accelerometer. PLoS One. 2011;6(7):e22922.

2. White T, Westgate K, Wareham NJ, Brage S. Estimation of Physical Activity Energy Expenditure during Free-Living from Wrist Accelerometry in UK Adults. PLoS One. 2016;11(12):e0167472.

3. Ainsworth BE, Haskell WL, Whitt MC, Irwin ML, Swartz AM, Strath SJ, et al. Compendium of physical activities: an update of activity codes and MET intensities. Medicine and science in sports and exercise. 2000;32(9 Suppl):S498-504.

4. Hildebrand M, VT VANH, Hansen BH, Ekelund U. Age group comparability of raw accelerometer output from wrist- and hip-worn monitors. Medicine and science in sports and exercise. 2014;46(9):1816-24.
