# Supplementary material for: The independent and joint association of accelerometer-measured physical activity and sedentary time with dementia: a cohort study in the UK Biobank
Source: Int J Behav Nutr Phys Act. 2023 May 17;20:59. doi: 10.1186/s12966-023-01464-8 (PMC10190060; doi:10.1186/s12966-023-01464-8)
Supplement: Supplementary file 4 — Additional file 4. Codes used in the UK Biobank study to identify dementia cases. [file 12966_2023_1464_MOESM4_ESM.docx]

**Additional File 4.** Codes used in the UK Biobank study to identify dementia cases.

| **ICD-9** | **ICD-10** | **Self-reported UK Biobank field code** |
| --- | --- | --- |
| AD: 331.0  VD: 290.4  Other codes for all-cause dementia:  290.2, 290.3, 291.2, 294.1, 331.1, 331.2, 331.5 | AD: F00, F00.0, F00.1, F00.2, F00.9,  G30, G30.0, G30.1, G30.8, G30.9  VD: F01, F01.0, F01.1, F01.2, F01.3,  F01.8, F01.9, I67.3  Other codes for all-cause dementia:  A81.0, F02, F02.1, F02.2, F02.3,  F02.4, F02.8, F03, F05.1, F10.6,  G31.0, G31.1, G31.8 | 1263 |

Abbreviations: ICD, International Classification of Diseases; AD, Alzheimer’s disease, VD, vascular dementia.
