# Supplementary material for: The independent and joint association of accelerometer-measured physical activity and sedentary time with dementia: a cohort study in the UK Biobank
Source: Int J Behav Nutr Phys Act. 2023 May 17;20:59. doi: 10.1186/s12966-023-01464-8 (PMC10190060; doi:10.1186/s12966-023-01464-8)
Supplement: Supplementary file 5 — Additional file 5. Detailed information on missing covariates. [file 12966_2023_1464_MOESM5_ESM.docx]

**Additional File 5.** Detailed information on missing covariates.

| **Variables** | **N*** | **Missing rate (%)** |
| --- | --- | --- |
| Ethnicity | 319 | 0.31 |
| Townsend deprivation index | 123 | 0.12 |
| Education | 1044 | 1.01 |
| Smoking status | 277 | 0.27 |
| Alcohol intake frequency | 87 | 0.08 |
| BMI category (kg/m^2^) | 243 | 0.23 |
| CVD event | 150 | 0.14 |
| Hypertension | 150 | 0.14 |
| Diabetes | 197 | 0.19 |
| Baseline depression | 4544 | 4.38 |
| History of cancer | 276 | 0.27 |

*N represents the number of missing responses.

Abbreviations: BMI, body mass index; CVD, cardiovascular disease.
