# Supplementary material for: The independent and joint association of accelerometer-measured physical activity and sedentary time with dementia: a cohort study in the UK Biobank
Source: Int J Behav Nutr Phys Act. 2023 May 17;20:59. doi: 10.1186/s12966-023-01464-8 (PMC10190060; doi:10.1186/s12966-023-01464-8)
Supplement: Supplementary file 6 — Additional file 6. Baseline characteristics of excluded individuals. [file 12966_2023_1464_MOESM6_ESM.docx]

**Additional File 6**. Baseline characteristics of excluded individuals.

| **Baseline characteristic** | **Overall** | **Excluded** | **Included** | ***P* value** |
| --- | --- | --- | --- | --- |
| **No.(%)** | 103,670 | 13,350 | 90,320 |  |
| **Follow-up, years, median (IQR)** | 6.9 (1.04) | 6.8 (1.0) | 6.9 (1.0) | **<0.001** |
| **TPA, milli-g, median (IQR)** | 26.74 (10.50) | 22.96 (18.82) | 27.08 (10.03) | **<0.001** |
| **Sedentary time, hours/day, median (IQR)** | 10.78 (2.33) | 11.62 (4.56) | 10.71 (2.19) | **<0.001** |
| **Age at baseline, years, mean (SD)** | 56.1 (7.8) | 55.4 (7.9) | 56.2 (7.8) | **<0.001** |
| **Sex, male, n (%)** | 45,396 (43.79) | 5,707 (42.75) | 39,689 (43.94) | **0.009** |
| **Ethnicity, n (%)** |  |  |  | **<0.001** |
| White | 95,284 (91.91) | 11,698 (87.63) | 83,586 (92.54) |  |
| Non-white | 8,067 (7.78) | 1,333 (9.99) | 6,734 (7.46) |  |
| Missing data, No.(%) | 319 (0.31) | 319 (2.39) | 0 |  |
| **Townsend deprivation index, n (%)** |  |  |  | **<0.001** |
| 1st quintile (least deprived) | 23,486 (22.65) | 2,658 (19.91) | 20,828 (23.06) |  |
| 2nd quintile | 22,371 (21.58) | 2,672 (20.01) | 19,699 (21.81) |  |
| 3rd quintile | 21,376 (20.62) | 2,741 (20.53) | 18,635 (20.63) |  |
| 4th quintile | 20,302 (19.58) | 2,668 (19.99) | 17,634 (19.52) |  |
| 5th quintile (most deprived) | 16,012 (15.45) | 2,488 (18.64) | 13,524 (14.97) |  |
| Missing data, No.(%) | 123 (0.12) | 123 (0.92) | 0 |  |
| **Education, n (%)** |  |  |  | **<0.001** |
| Low | 8,464 (8.16) | 1,259 (9.43) | 7,205 (7.98) |  |
| Intermediate | 49,528 (47.77) | 6,195 (46.40) | 43,333 (47.98) |  |
| High | 44,634 (43.05) | 4,852 (36.34) | 39,782 (44.05) |  |
| Missing data, No.(%) | 1,044 (1.01) | 1,044 (7.82) | 0 |  |
| **Smoking status, n (%)** |  |  |  | **<0.001** |
| Never | 58,956 (56.87) | 7,317 (54.81) | 51,639 (57.17) |  |
| Previous | 37,162 (35.85) | 4,679 (35.05) | 32,383 (35.96) |  |
| Current | 7,275 (7.02) | 1,077 (8.07) | 6,198 (6.86) |  |
| Missing data, No.(%) | 277 (0.27) | 277 (2.07) | 0 |  |
| **Alcohol intake frequency, n (%)** |  |  |  | **<0.001** |
| Never | 5,870 (5.66) | 859 (6.43) | 5,011 (5.55) |  |
| Less than 3 times/week | 47,009 (45.34) | 6,332 (47.43) | 40,677 (45.04) |  |
| ≥ 3 times/week | 50,704 (48.91) | 6,072 (45.48) | 44,632 (49.42) |  |
| Missing data, No.(%) | 87 (0.08) | 87 (0.65) | 0 |  |
| **BMI category (kg/m2), n (%)** |  |  |  | **<0.001** |
| Underweight (<18.5) | 583 (0.56) | 80 (0.60) | 503 (0.56) |  |
| Normal weight (18.5 to <25) | 39,986 (38.57) | 4,799 (35.95) | 35,187 (38.96) |  |
| Overweight (25 to <30) | 42,571 (41.06) | 5,315 (39.81) | 37,256 (41.25) |  |
| Obese >=30* | 20,287 (19.57) | 2,913 (21.82) | 17,374 (19.24) |  |
| Missing data, No.(%) | 243 (0.23) | 243 (1.82) | 0 |  |
| **CVD event, n (%)** | 4,059 (3.92) | 571 (4.28) | 3,488 (3.86) | **0.010** |
| Missing data, No.(%) | 150 (0.14) | 150 (1.12) | 0 |  |
| **Hypertension, n (%)** | 23,720 (22.88) | 3,108 (23.28) | 20,612 (22.82) | 0.064 |
| Missing data, No.(%) | 150 (0.14) | 150 (1.12) | 0 |  |
| **Diabetes, n (%)** | 3,580 (3.45) | 514 (3.85) | 3,066 (3.39) | **0.003** |
| Missing data, No.(%) | 197 (0.19) | 197 (1.48) | 0 |  |
| **Baseline depression, n (%)** | 3,934 (3.79) | 458 (3.43) | 3,476 (3.85) | 0.644 |
| Missing data, No.(%) | 4,544 (4.38) | 4,544 (34.04) | 0 |  |
| **History of cancer, n (%)** | 7,593 (7.32) | 973 (7.29) | 6,620 (7.33) | **<0.001** |
| Missing data, No.(%) | 276 (0.27) | 276 (2.07) | 0 |  |
| **APOE ε4 carrier status, n (%)** |  |  |  | 0.391 |
| ε4 non-carrier | 62,194 (59.99) | 7,998 (59.91) | 54,196 (60.00) |  |
| One ε4 allele | 22,385 (21.59) | 2,803 (21.00) | 19,582 (21.68) |  |
| Two ε4 alleles | 1,915 (1.85) | 251 (1.88) | 1,664 (1.84) |  |
| Missing data, No.(%) | 17,176 (16.57)) | 2,298 (17.21) | 14,878 (16.47) |  |

*Includes World Health Organization obese and obesity classes 2 and 3 categories.

Abbreviations: TPA, total volume of physical activity, milli-g; BMI, body mass index; CVD, cardiovascular disease; IQR, interquartile range; SD, standard deviation
