# Supplementary material for: The independent and joint association of accelerometer-measured physical activity and sedentary time with dementia: a cohort study in the UK Biobank
Source: Int J Behav Nutr Phys Act. 2023 May 17;20:59. doi: 10.1186/s12966-023-01464-8 (PMC10190060; doi:10.1186/s12966-023-01464-8)
Supplement: Supplementary file 7 — Additional file 7. Association of TPA and sedentary time with dementia excluding dementia onset within 2 years (n = 47). [file 12966_2023_1464_MOESM7_ESM.docx]

**Additional File 7. Association of TPA and sedentary time with dementia excluding dementia onset within 2 years (n=47).**

| **Groups** | | **Events/N** | **Incidence rate (%)** | **HR (95% CI)** | ***P* value** | **Additive interaction (RERI)** |  |  | **Multiplicative interaction** |  |
| --- | --- | --- | --- | --- | --- | --- | --- | --- | --- | --- |
|  |  |  |  |  |  | **Estimates (95% CI)** | ***P* value** |  | **Estimates (95% CI)** | ***P* value** |
| **TPA, milli-g** | |  |  |  |  |  |  |  |  |  |
| Low | | 332/45143 | 0.74 | 1.00 (Ref) |  |  |  |  |  |  |
| High | | 122/45130 | 0.27 | **0.60 (0.49, 0.75)** | **<0.001** |  |  |  |  |  |
| **Sedentary time, h/ day** | |  |  |  |  |  |  |  |  |  |
| Low | | 181/45415 | 0.40 | 1.00 (Ref) |  |  |  |  |  |  |
| High | | 273/44858 | 0.61 | **1.28 (1.06, 1.55)** | **0.012** |  |  |  |  |  |
| **TPA, milli-g** | **Sedentary time, h/ day** |  |  |  |  |  |  |  |  |  |
| High | Low | 100/34362 | 0.29 | 1.00 (Ref) |  |  |  |  |  |  |
| High | High | 22/10768 | 0.20 | 0.82 (0.52, 1.30) | 0.404 |  |  |  |  |  |
| Low | Low | 81/11053 | 0.73 | **1.47 (1.09, 1.98)** | **0.012** |  |  |  |  |  |
| Low | High | 251/34090 | 0.74 | **1.64 (1.29, 2.10)** | **<0.001** | 0.35 (-0.17, 0.88) | 0.188 |  | 1.43 (0.85, 2.42) | 0.181 |

Models were adjusted for age at baseline, sex, ethnicity, education and Townsend deprivation index, smoking status, alcohol intake frequency, body mass index (BMI), history of cardiovascular disease (CVD), hypertension, diabetes, cancer and depression.

Abbreviations: TPA, total volume of physical activity; milli-g; RERI, relative excess risk due to interaction.
