# Supplementary material for: The independent and joint association of accelerometer-measured physical activity and sedentary time with dementia: a cohort study in the UK Biobank
Source: Int J Behav Nutr Phys Act. 2023 May 17;20:59. doi: 10.1186/s12966-023-01464-8 (PMC10190060; doi:10.1186/s12966-023-01464-8)
Supplement: Supplementary file 10 — Additional file 10. Association of TPA and sedentary time with dementia using a 9.5-h cutoff for sedentary time. [file 12966_2023_1464_MOESM10_ESM.docx]

**Additional File 10. Association of TPA and sedentary time with dementia using a 9.5-hour cutoff for sedentary time.**

| **Groups** | | **Events/N** | **Incidence rate (%)** | **HR (95% CI)** | ***P* value** | **Additive interaction (RERI)** |  |  | **Multiplicative interaction** |  |
| --- | --- | --- | --- | --- | --- | --- | --- | --- | --- | --- |
|  |  |  |  |  |  | **Estimates (95% CI)** | ***P* value** |  | **Estimates (95% CI)** | ***P* value** |
| **TPA, milli-g** | |  |  |  |  |  |  |  |  |  |
| Low | | 369/45180 | 1.20 | Ref. |  |  |  |  |  |  |
| High | | 132/45140 | 0.43 | **0.35 (0.29, 0.43)** | **<0.001** |  |  |  |  |  |
| **Sedentary time, h/ day** | |  |  |  |  |  |  |  |  |  |
| Low (<9.5) | | 89/20,848 | 0.62 | Ref. |  |  |  |  |  |  |
| High (≥ 9.5) | | 412/69,472 | 0.87 | 1.15 (0.91, 1.22) | 0.244 |  |  |  |  |  |
| **TPA, milli-g** | **Sedentary time, h/ day** |  |  |  |  |  |  |  |  |  |
| High | Low | 62/18,517 | 0.49 | Ref. |  |  |  |  |  |  |
| High | High | 70/26,623 | 0.38 | 0.86 (0.61, 1.21) | 0.381 |  |  |  |  |  |
| Low | Low | 27/2,331 | 1.71 | **1.90 (1.20, 3.00)** | **0.006** |  |  |  |  |  |
| Low | High | 342/42,849 | 1.18 | **1.57 (1.19, 2.07)** | **0.002** | -0.19 (-1.01, 0.63) | 0.649 |  | 0.96 (0.57, 1.62) | 0.883 |

Models were adjusted for age at baseline, sex, ethnicity, education and Townsend deprivation index, smoking status, alcohol intake frequency, body mass index (BMI), history of cardiovascular disease (CVD), hypertension, diabetes, cancer and depression.

Abbreviations: TPA, total volume of physical activity; milli-g; RERI, relative excess risk due to interaction.
